# Endoscopy Assessment at 1-Year Identifies Long-Term Responders to Thiopurines Maintenance Therapy in Patients With Crohn's Disease

**DOI:** 10.1097/MD.0000000000001204

**Published:** 2015-08-07

**Authors:** Yun Qiu, Bai-li Chen, Ren Mao, Sheng-hong Zhang, Yao He, Zhi-rong Zeng, Min-hu Chen

**Affiliations:** From the Department of Gastroenterology, The First Affiliated Hospital of Sun Yat-Sen University, Guangzhou, People's Republic of China.

## Abstract

When treating Crohn disease (CD) with thiopurines, achievement of an objective response is essential. However, the minimal degree of mucosal improvement required to alter disease outcomes of CD is unknown.

To determine the endoscopic responses of thiopurine monotherapy and to determine the minimal degree of mucosal improvement required to alter disease outcomes of CD.

One hundred thirty CD patients who had evaluable ileocolonoscopy with evident of mucosal ulceration at baseline were included. The endpoints were endoscopic responses at the 2 follow-up endoscopies performed at 12 months (M12) and 36 month (M36) from the initiation of thiopurines.

At M12, mucosal healing (MH) and a positive endoscopic response (PR) were documented in 38% and 46% of patients, respectively. At the second follow-up, merely a further 14% (13/93) of patients on monotherapy had a PR and a total of 46% (43/93) presented with MH. In a Cox regression model, both a PR (*P* < 0.02) and MH (*P* < 0.001) at M12 were associated with response at M36 in patients continuing thiopurine treatment. MH at M12 was associated with long-term disease outcomes of CD at M36, with an area under the Receiver Operator Characteristic curve of 0.54 for predicting clinical remission, 0.69 for hsCRP normalization, 0.69 for MH, and 0.74 for PR, respectively. A PR at M12, defined as a decrease in endoscopic activity score by ≥2 points from baseline, yielded similar results.

Endoscopy at M12 can help to identify responders to thiopurine monotherapy in active CD. A PR could represent the minimal clinically important improvement in endoscopic disease activity.

## INTRODUCTION

Thiopurine have been associated with both clinical improvement and mucosal healing (MH) in treating Crohn disease (CD), even though it is well known that this drug takes a relatively long time to achieve its potential benefits.^1–4^ Unfortunately, the high rate of adverse events leading to drug withdrawal represents a major limitation in the use of these drugs.^5^ Long-term thiopurine therapy fails in approximately 50% patients who experience significant toxicity or inadequate response during treatment.^6^ Thus, when treating CD with thiopurine, achievement of an objective treatment response is essential. This also fits the future research agenda proposed by the Thiopurine Task Force Interest Group to identify patients who will benefit from thiopurine therapy to prevent disease recurrence.^7^

Accumulating evidence point that MH may change the natural course of the disease by decreasing rates of clinical relapse, CD-related hospitalization, and the need for surgery.^8–10^ However, little is known the minimal degree of endoscopic improvement needed to achieve such benefits. In a retrospective study, complete as well as partial MH was associated with a significantly lower need for major abdominal surgery.^9^ A subgroup analysis of patients from the Study of Biologic and Immunomodulator Naive Patients in Crohn's Diseases (SONIC trial) indicated that endoscopic response, defined as a decrease from baseline in the Simple Endoscopic Score for Crohn's Disease (SES-CD) or the Crohn's Disease Endoscopic Index of Severity (CDEIS) score of at least 50% at week 26, predicted corticosteroid free clinical remission (CFREM) at week 50.^11^

Till now, only a limited amount of data on the effect of thiopurine on MH were available from endoscope guided studies,^12^ which long before trials with biological therapy. No clear cut-off value of endoscopic activity that represents the minimal clinically significant improvement has been identified.

The aims of this retrospective study were to determine the endoscopic responses for thiopurine maintenance therapy and to evaluate the role of endoscopy in predicting long-term response to thiopurine in active CD.

## METHODS

### Patients and Design

This was an observational study of a sing-center cohort. All consecutive patients with a diagnosis of CD who received AZA/6-MP treatment at the Gastroenterology Outpatient Clinic of the First Affiliated Hospital of Sun Yat-Sen University between 2000 and 2014 were included. Diagnoses of CD were established according to the criteria of Lennard-Jones,^13^ and location of disease was made according to the criteria of the Montreal Classification.^14^

AZA/6-MP was given to all CD patients who fulfilled a set of criteria: moderate to severely active ileocecal or colonic CD; clinical factors that suggested a poor prognosis (diagnosis before 40 years of age, perianal disease, extensive involvement of the colon, and deep ulceration); steroid dependency or extensive small bowel or esophageal/gastroduodenal involvement.

The inclusion criteria for the study were patients aged ≥16 years old; ulcers detected by (ileo) colonoscopy at the initial endoscopy procedure; who had ≥2 consecutive endoscopic procedures performed during the study period; patients who received thiopurines ≥6 months; the concentration of 6-thioguanine nucleotide (6-TGN) within the target therapeutic window.

Exclusion criteria of this study were patients with incomplete endoscopic procedures; aged <16 years; isolate upper gastrointestinal tract or small bowel involvement at the time of diagnosis according to the Montreal classification^14^; the introduction of biotherapy or methotrexate or long-term steroid (prednisone or budesonide) during the AZA/6-MP treatment; an immediate need for surgery; contraindication to thiopurines according to labeling recommendations.

The study protocol was approved by the Clinical Research Ethics Committee of The First Affiliated Hospital of Sun Yat-Sen University.

### Treatment Schedules: Dosing and Duration

According to the major available guidelines^15–17^ AZA dose was targeted at 2.0 to 2.5 mg/kg body weight and 6-mercaptopurine (6-MP) at 1.0 to 1.5 mg/kg body weight by regular monitoring the 6-TGNs concentrations to achieve the therapeutic window of 250 to 400 pmol/8 × 10^8^ erythrocyte.

### Clinical Follow-Up and Data Collection

The clinical follow-up and other relevant data in the medical files of the patients were reassessed by 2 experienced gastroenterologists (MHC and BLC). A predetermined structured data sheet was used to collect data from the medical files, including: general well-being, symptoms of the disease before and during the thiopurines medication, thiopurines initiation dates and dosage, and comedication. The incidence of CD-related hospitalization, perianal surgery, intestinal surgery, median of the Crohn's Disease Activity Index (CDAI) scores and C-reactive protein (CRP) concentrations at the successive visits throughout patient follow-up were registered.

### Endoscopic Follow-Up

The endoscopy reports were recorded in the patients’ pro forma questionnaire sheet and also saved as a digital version in the endoscopy registry. The endoscopic scored system was adopted from Björkesten et al,^18^ which is a semi-quantitative scores that ranged from 0 to 6 based on the severity of inflammatory activity. The scores were based on consensus of the 2 specialists (BLC and YH) unblinded. The numbers of patients with a positive response (PR), a negative response (NR), and a MH were recorded at the time of each endoscopic procedure.

### Definitions

The primary endpoint for the efficacy of thiopurine treatment was evaluated at the first follow-up endoscopy at M12 and the secondary endpoint was the second follow-up endoscopy at M36 from the commencement of thiopurine treatment. CFREM was defined as the absence of flare, with no corticosteroid or anti-TNF use, no active perianal disease, no hospitalization related to CD, and no surgical procedures. Flare was defined by a CDAI score >150 or an increase in CDAI of ≥70 points. Biological response at M12 was evaluated in the subgroup of 88 patients with an elevated hsCRP level (≥3 mg/L) at inclusion. Biological response was defined as a normalization of hsCRP level (<3 mg/L). The criterion for PR was a decrease in the endoscopic score of ≥2 points, and a decrease of <2 points was considered as NR.^18^ MH was defined as a mucosal activity score of 0 to 2.^9^

### Statistical Analysis

Demographic and clinical parameters were compiled and summary statistics calculated. Data were described using medians with interquartile range (IQR) for continuous data and percentages for discrete data. For statistical analysis Fisher exact test and Chi square tests were used to compare the nonparametric categorical data between groups and analysis of variance (ANOVA) for continuous parameters. We used Cox regression analysis to evaluate risk factors of endoscopic response and CFREM. Factors analyzed by univariate analysis with *P* < 0.1 were integrated in multivariate Cox regression. Time-to-event analysis was performed with the Kaplan–Meier curve.

The endoscopic and/or biomarker remission at M36 were used as a binary classifier to evaluated the diagnostic ability of endoscopic activity and biological activity also CFREM at M12 by calculating sensitivity, specificity, positive likelihood ratios (PLR), negative likelihood ratios (NLR), and receiver operating characteristic (ROC) curves with the 95% confidence intervals (CIs). Of note, the NLR/PLR corresponds to the likelihood of (no) MH at M12 in patients with CFREM at M36 relative to that in patients without CFREM at M36.^19^ For comparison, the association between each evaluated parameter at M12 (CFREM, biomarkers, and endoscopic findings) and endoscopic healing and/or clinical/biomarker remission at M36 was also evaluated, provided with a *P*-value (Pearson χ^2^).

The SPSS 15.0 software (SPSS, Chicago, IL) and MedCalc software, V.11.6.1.0 (MedCalc Software, Belgium) were used to perform all appropriate statistical analyses. For all tests, statistical significance was set at *P* < 0.05.

## RESULTS

### Study Population

Baseline endoscopy (ileocolonoscopy) was performed on all 268 patients. A total of 130 patients who had evidence of mucosal ulcerations at baseline and had evaluable ileocolonoscopy, CDAI and hsCRP values at M12 were included in the analysis, among which 88 patients with hsCRP ≥3 mg/L (Table 1). Demographic characteristics of the 130 patients included in this analysis were comparable with those of the 138 patients who were excluded. All patients presented with moderate to very severe luminal inflammation as determined by endoscopy (Björkesten mucosal activity score of 4–6).

**TABLE 1:**
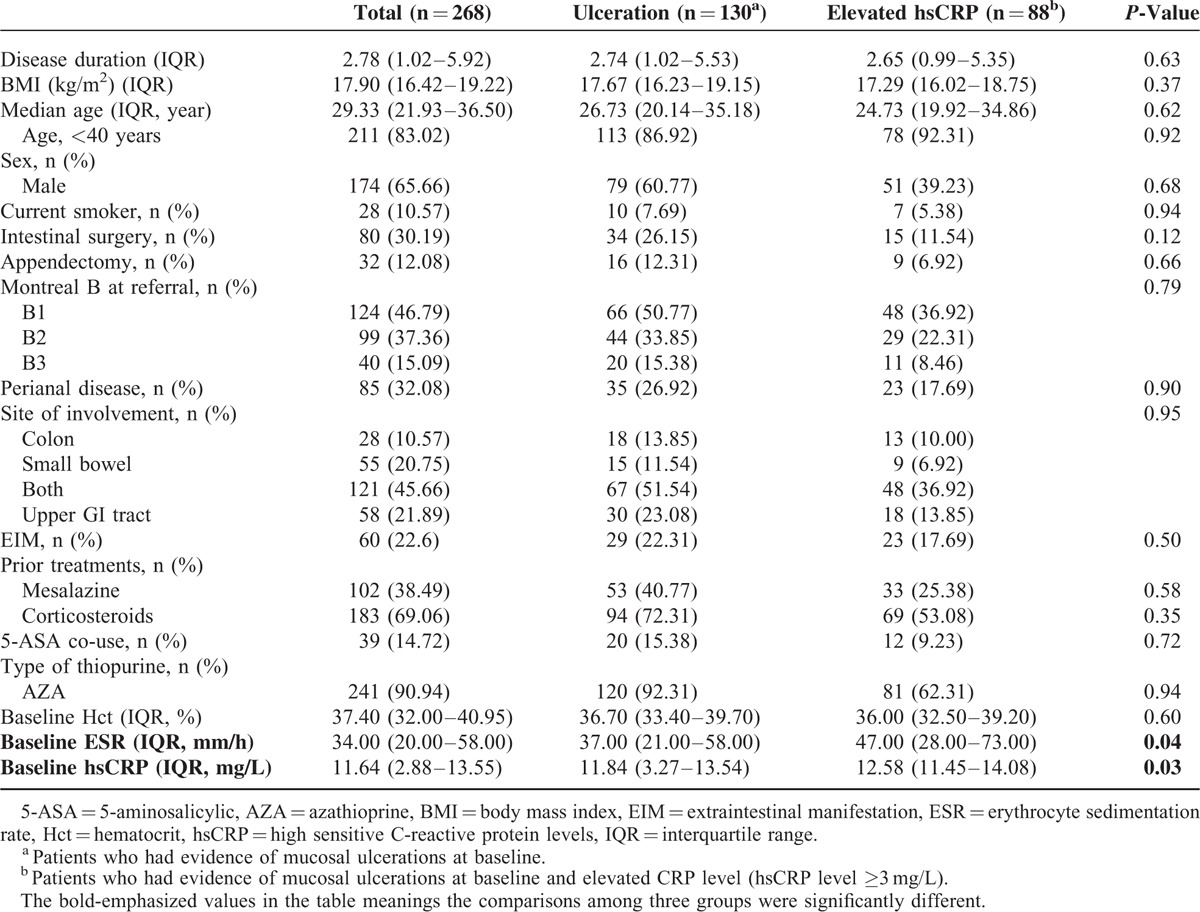
Baseline Characteristics of the Patients Included (n = 130) or Excluded (n = 138) From This Analysis

Thiopurine treatment was started within a median of 0.6 month from baseline endoscopy. The outcome of thiopurine treatment was mainly evaluated by 2 follow-up endoscopies at about 12 months (median 11.1, IQR, 7.8–13.1 months) from the start of thiopurine therapy and at about 36 months (median 36.8, IQR, 32.1–40.6 months).

### First Follow-Up Endoscopy, 12 Months From Start of Thiopurine

**FIGURE 1:**
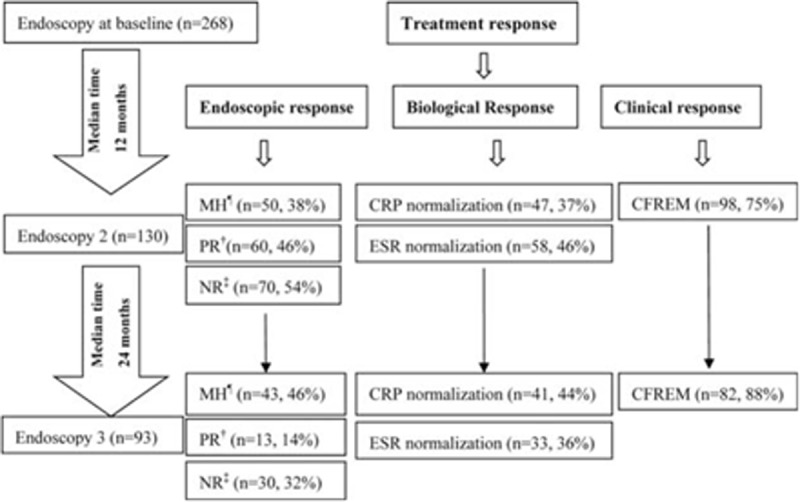
Flow chart of clinical, endoscopic findings and biological response during the study period. ^†^Positive response was defined as a decrease in endoscopic activity score by at least 2 points from baseline; ^‡^Negative response was defined as a decrease in endoscopic activity score by less than 2 points. ^¶^Mucosal healing was defined as mucosal activity score 0 to 2, that is, no inflammatory activity or only mild inflammation without ulcerations.

Ileocolonoscopy revealed that 46% (60/130) of the patients had a significant improvement in inflammatory activity, and 38% (50/130) of the patients had MH. CFREM were achieved at M12 in 98 (75%) patients (Figure 1). Complete and partial biological responses were achieved by 37% and 75% of patients at M12 in 88 patients with elevated hsCRP levels at baseline, respectively.

### Factor Associated With Endoscopic Outcomes

**FIGURE 2:**
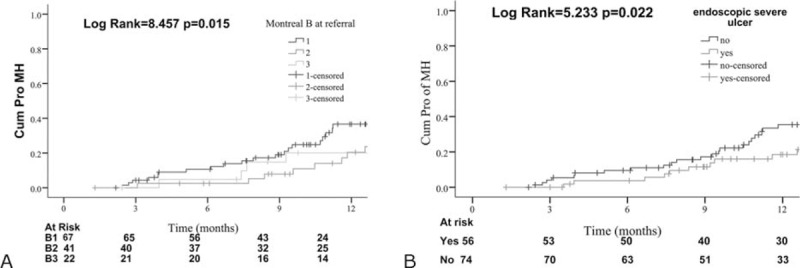
Kaplan–Meier analysis of reaching MH at M12 according to factors identified by multivariate analysis: (A) Montreal B at CD diagnosis; (B) endoscopic procedures within 26 weeks.

For predictors of endoscopic outcomes, all baseline factors were evaluated in univariate analysis using the Mantel–Cox log-rank test (Table 2). Significant factors predicted MH was naïve to 5-ASA use (HR, 2.16; 95% CI, 1.11–4.18, Table 2). Conversely, Montreal B3 at referral (HR, 0.54; 95% CI, 0.35–0.83) and baseline severe ulcer (HR, 0.49; 95% CI, 0.27–0.91) were negatively associated with MH. In multivariate analysis, Montreal B3 at referral (adjusted HR, 0.51; 95% CI, 0.33–0.80) and baseline severe ulcers (adjusted HR, 0.44; 95% CI, 0.24–0.83) independently predicted MH (Table 2, Figure 2).

**TABLE 2:**
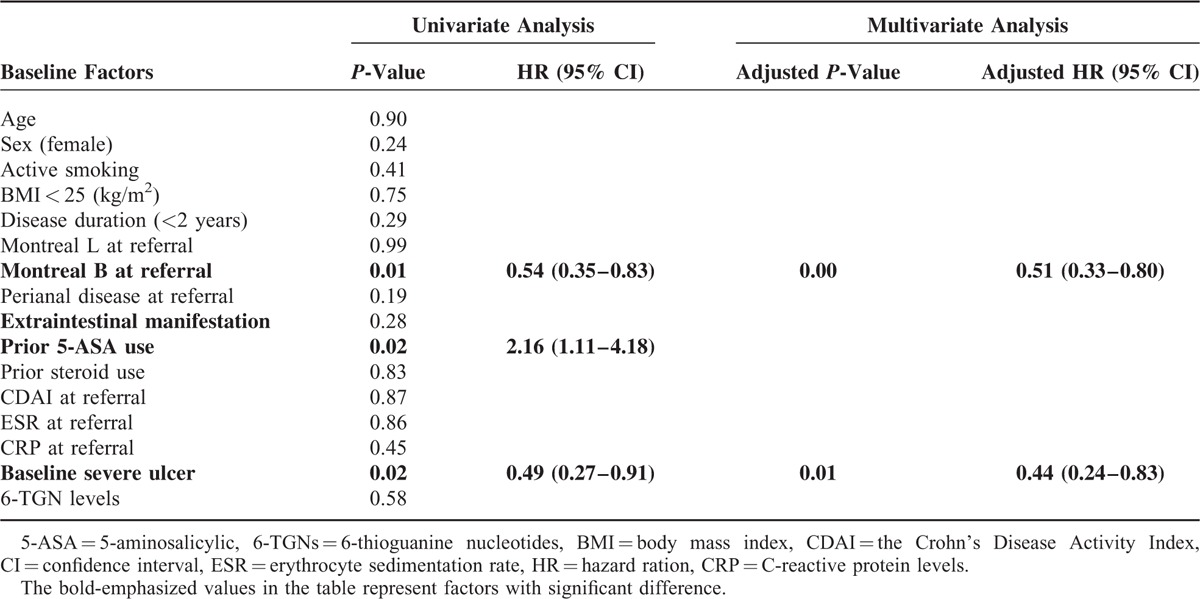
Predictors of Mucosal Healing by Univariate Analysis (Log-Rank Test) and by Univariate (Cox Model) at 12 Months

The same baseline factors were analyzed to identify predictors of endoscopic improvement. A PR treatment response at M12 was negatively associated with Montreal B3 at referral (adjusted HR, 0.54; 95% CI, 0.35–0.81) but not with the baseline endoscopy score (adjusted HR, 0.97; 95% CI, 0.76–1.23; Supplementary Table 1).

### Second Follow-up Endoscopy at M36 After Initiating Thiopurine

Thirty-seven patients discontinued thiopurine during the subsequent 2-year follow-up, 15 patients because of AE, 11 patients due to inefficacy (9 with frequent relapses, 2 undergone operations), and another 11 patients stopped with arbitrary reason (safety concerns, pregnancy, poor compliance, etc.). Further endoscopic data were available for 93 of the original 130 patients at M36. Figure 1 demonstrates data of the further 2-year follow-up endoscopic evaluations of the initial responders and nonresponders on continuation of thiopurine treatment. At the second follow-up, merely a further 14% (13/93) of patients on monotherapy had a PR and a total of 46% (43/93) presented with MH. Endoscopic findings, particularly for those of MH at the first follow-up endoscopy, were strongly associated with their persistence at the second follow-up endoscopy, maintained in 86% of patients when thiopurine was continued (r = 0.403, *P* = 0.002). On the contrary, if the initial endoscopic response was negative, the long-term response remained poor. However, neither a clinical response nor a biological response at M12 was significantly associated with endoscopic findings at M36.

### Factor Associated With Endoscopic Outcomes

By using multivariate analysis, none of the baseline factors independently predicted MH at M36 (Supplementary Table 2). PR at M12 (HR, 3.39; 95% CI, 1.51–7.58) and MH at M12 (HR, 2.93; 95% CI, 1.32–6.5) were positively associated with MH at M36 (Table 3). Conversely, CDAI at M12 (HR, 0.99; 95% CI, 0.98–1), CRP at M12 (HR, 0.8; 95% CI, 0.69–0.93), and endoscopy score at M12 (HR, 0.77; 95% CI, 0.62–0.95) were associated negatively with MH at M36 (Table 3). Multivariate analysis showed that a PR at M12 independently predicted MH at M36 (HR, 3.54; 95% CI, 1.54–8.16) (Table 3). However, with regard to endoscopic improvement, no significant predictor was identified.

**TABLE 3:**
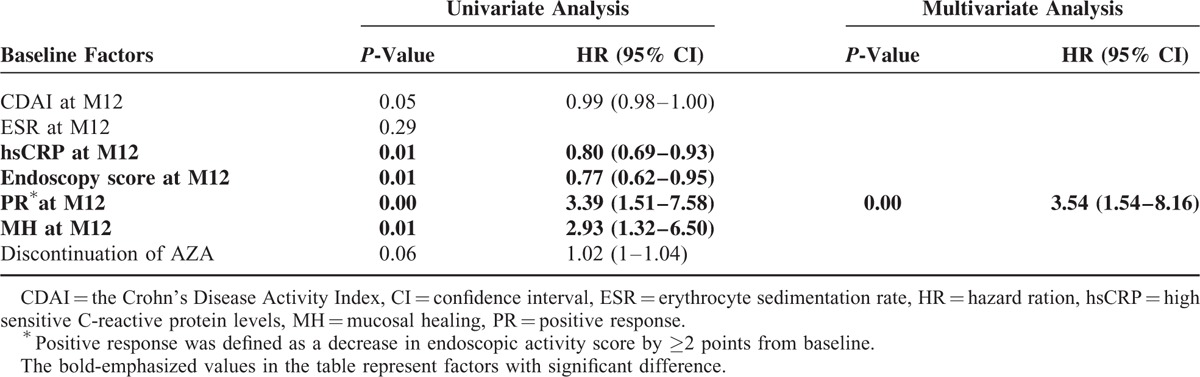
Predictors of Mucosal Healing at M36 by Univariate Analysis (Log-Rank Test) and by Multivariate (Cox Model) Analysis

Additionally, ROC curves were constructed to assess the power of disease activity markers at M12 to predict long-term outcomes in patients receiving thiopurine maintenance therapy. The achievement of MH at M12 had the best overall performance for predicting both the biological response and endoscopic response at M36, with an AUC of 0.69 (predictive of hsCRP normalization at M36; standard error (SE), 0.07) and 0.74 (predictive of PR at M36; SE, 0.04), 0.69 (predictive of MH at M36; SE, 0.06), respectively. The achievement of PR at M12 had a comparable capacity with MH for predicting both the biological response and endoscopic response (Table 4).

**TABLE 4:**
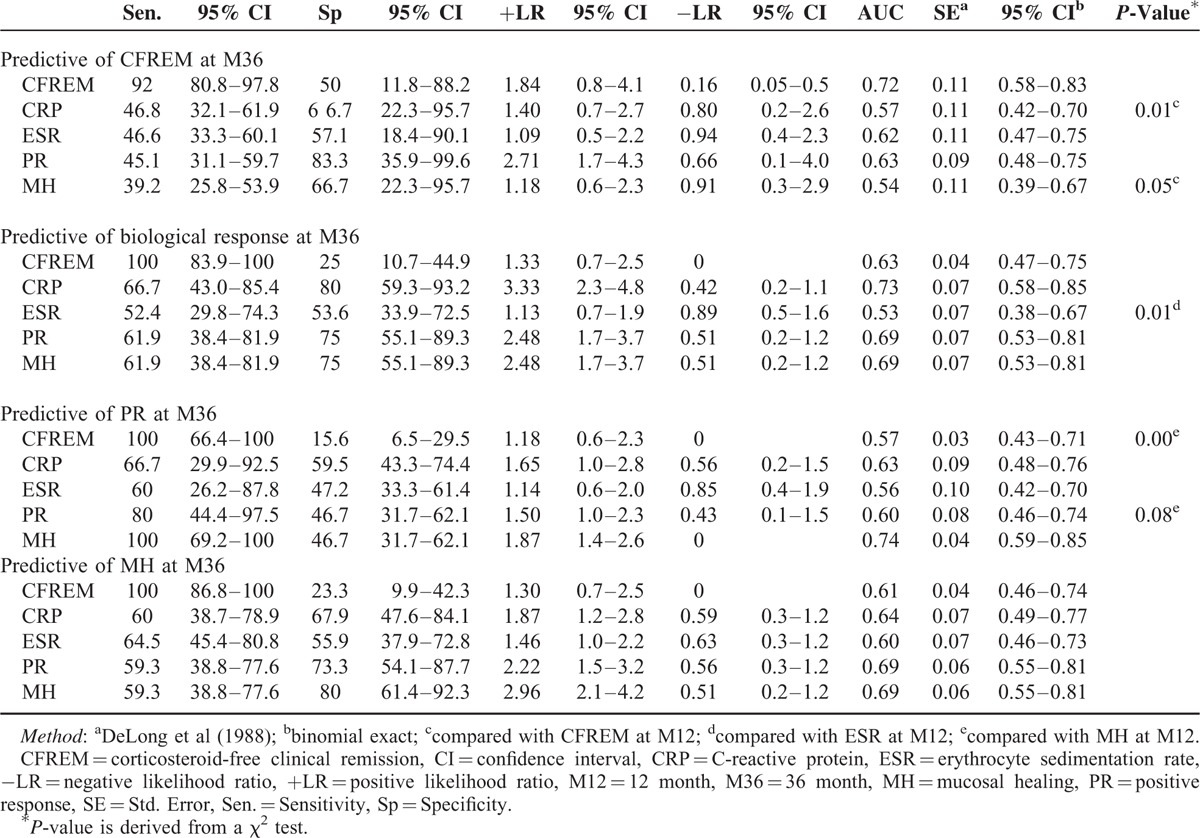
Comparison of ROC Curves for Predictive of CFREM or PR or MH at 36 Month Using the Clinical Response (CFREM) or Endoscopic Response (PR or MH) or Biological Response (ESR, hsCRP) at 12 Months

Whereas, MH at M12 only had a minimal capacity for predicting CFREM at M36, with a sensitivity of 0.39 (95% CI, 0.26–0.54), a specificity of 0.67 (95% CI, 0.22–0.96), a PLR of 1.18 (95% CI, 0.6–2.3), a NLR of 0.91 (95% CI, 0.3–2.9), and an AUC of 0.54 (SE, 0.11), respectively. On the contrary, PR at M12 had an improved performance for predicting CFREM at M36 with an AUC of 0.63 (SE, 0.09) (Table 4).

### Long-Term Outcomes

Few prospective data were available to support the clinical relevance of MH in patients with CD. Our study further examined whether complete healing, determined by endoscopy, predicted a better outcome in CD. Both MH and a PR at M12, predicted sustained CFREM 3 years after thiopurine initiation. The mean time span of CFREM among patients who achieved MH was 49.2 ± 3.7 months compared to 44.2 ± 4.2 months of patients without achieving MH (*P* = 0.02). However, there was only a trend of longer mean time span of CFREM (45.3 ± 4.6 months) among patients who achieved PR, albeit not significant different, when compared to 41.9 ± 5.4 months of the NR group (*P* = 0.14).

### Surgical Intervention and Hospitalizations

Sixteen (16.1%) patients required hospitalization due to disease flare (7 (6.1%) patients in the PR group and 9 (10.03%) patients in the NR group, Table 5). Due to persisting disease activity and strictures, 8 patients (5 (9.13%) patients in the PR group and 3 (14.9%) patients in the NR group) underwent surgical procedures after initiation of thiopurine treatment. No significant differences in the rates of adverse events were observed between patients with MH (19, 7.8%) and without MH (32, 7.1%, Table 5).

**TABLE 5:**
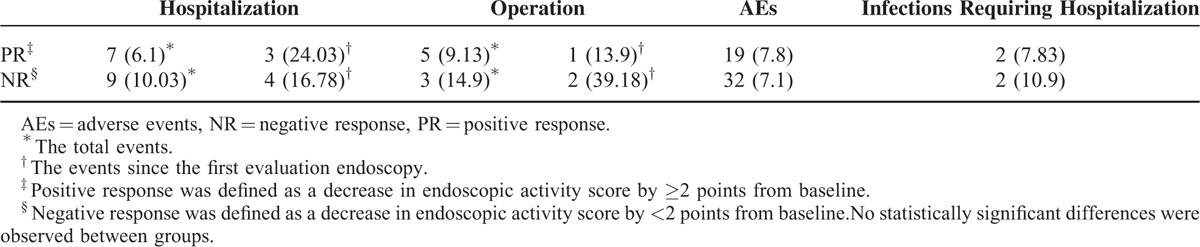
Adverse Events, Comorbidities, and Hospitalizations

## DISCUSSION

MH, defined as a complete absence of mucosal ulcerations, was documented in 38% of the patients at the first follow-up endoscopy. When thiopurine was continued in patients with objective initial response, MH was maintained in the majority (86%) of patients with CD. Thus, MH at 1-year may serve as an objective treatment response for patients with CD on thiopurine monotherapy. On the contrary, the discrepancy between CDAI and endoscopic findings (r = −0.04; *P* = 0.66) or between hsCRP and endoscopic findings (r = 0.15; *P* = 0.13) also confirmed in our cohort.

MH is receiving increasing attention based on observations that treatment aiming at clinical symptoms resolution alone does not prevent long-term bowel damage. In the present study, MH was clearly predictive of sustained clinical benefit at M36. So far no controlled prospective trials designed exclusively to identify predictors of MH have been conducted, although this is highly desirable given the toxicity of prolonged immunomodulation. A subgroup analysis of the recent EXTEND trial showed a higher rate of MH among patients who received Adalimumab and had a CD duration shorter than 2 years,^20^ however, it failed confirmed by a recent study.^21^ The CRP level at time of endoscopy was significantly correlated with the degree of MH.^22^ Kiss et al^23^ also suggested CRP at week 12, clinical remission at week 24 were associated to endoscopic improvement/healing during the first year of Adalimumab therapy. However, in the present study, we failed to demonstrate a significant association between disease duration or CRP level and the subsequent MH response. According to our study, a MH treatment response at M12 was significantly associated to penetrating disease behavior (Montreal B3) at referral (adjusted HR, 0.51), and baseline endoscopy score (adjusted HR, 0.44) by multivariate analysis. Similarly, a PR treatment response at M12 was also significantly associated to Montreal B3 at referral. Penetrating disease behavior at diagnosis and severe lesions as clinical predictors of aggressive/disabling disease already had been previously reported.^24^

However, complete MH is only achieved by a minority of patients. In the present study, MH was documented in 38% of the patients at the first follow-up endoscopy. A subgroup analysis using data of SONIC trial indicate that endoscopic response, when defined as a decrease from baseline in SES-CD or CDEIS score of ≥50% at week 26, predicted CFREM at week 50 and therefore could be proposed as an cut-off value for endoscopic response.^11^ Therefore, we evaluated whether endoscopic response could serve as a more practical therapeutic goal predicting sustained clinical response in patients receiving thiopurine therapy.

Importantly, a PR which defined as a decrease from baseline in endoscopic score of at least 2 points at M12 had a comparable value with MH for predicting the beneficial clinical effect at M36 using both the clinical and endoscopic endpoints according to our study. Therefore, achieving such an endoscopic response at M12, a less stringent end point, may be sufficient to alter the clinical outcome of CD. Patients with NR at M12 might benefit from early treatment optimization. The well-established evidence support that maintenance of MH at M36 was predictive of other desired disease modification benefits such as the prevention of bowel damages. Thus, although due to the relative low incidence of hospitalization and intestinal surgery, we did not demonstrate such benefits in our cohort, it was promising such benefits exists due to its nature of disease modification. The achievement of endoscopic response was a reliable predictor for long-term outcome (sustained clinical response, surgery-free survival). Further validation in an independent prospective cohort with end points on disease course is still required.

Our preliminary study results should be considered with caution for several reasons. First, the retrospective design could induce a bias of patient selection and a bias of data gathering. However, the present study cohort of patients was a representative sample of consecutive enrolment of the patients referred to our IBD Center. Most of the data (from year 2003 to 2014) were collected prospectively and the clinical symptoms, biological response and endoscopic findings had been structurally documented in the patients’ medical files during each follow-up which made these biases minimal. Second, the lack of a validated endoscopic scoring system was an important limitation. Our previous study demonstrated the Björkesten score correlate well with the SES-CD (r = 0.743) or CDEIS score (r = 0.738) (data not published). Actually, due to the lack of a standard criteria for MH, the definition we adopted was consisted with that used by Frøslie et al,^8^ in which normal endoscopic findings to light mucosal erythema or granularity without ulcerations were all regarded as MH. Third, the varying time intervals between endoscopies were another limitation. But the majority of the patients underwent both baseline endoscopy within a month before the initiation of thiopurine and the subsequent follow-up endoscopies performed around 9 to 13 and 32 to 40 months. Fourth, compliance to thiopurine treatment was not assessed. But regular checks of 6-TGN concentration were within the target therapeutic window. Last but not the least, we reported a high rate of drug withdraw regarding long-term outcomes, 28% of patients discontinued AZA/6-MP. However, multivariate analysis confirmed the discontinuation of thiopurine was not a risk factor for loss of MH.

Taken together, our preliminary study results indicated that in patients with CD on thiopurine monotherapy, MH and endoscopic response at M12 could identify those most likely to achieve an endoscopic response at M36. The correlation between the proposed endoscopic response and changes in long-term disease progression, evident as a lower risk of CD-related surgeries and hospitalizations, still needs to be demonstrated.
